# Comprehensive evaluation of root and root canal morphology of mandibular second molars in a Saudi subpopulation evaluated by cone-beam computed tomography

**DOI:** 10.1186/s12903-022-02305-z

**Published:** 2022-07-01

**Authors:** Moazzy I. Almansour, Saad M. Al‑Zubaidi, Abdulmjeed S. Enizy, Ahmed A. Madfa

**Affiliations:** 1 Department of Restorative Dental Science, Collage of Dentistry, University of Ha’il, Ha’il, Kingdom of Saudi Arabia; 2 Department of Dentistry, Prince Sultan Military Medical City, Riyadh, Saudi Arabia

**Keywords:** Cone beam computed tomography, C-shaped root canal system, Endodontics, Mandibular second molar, Root canal anatomy, Saudi subpopulation

## Abstract

**Background:**

The study's goal was to use Cone Beam Computed Tomography (CBCT) to assess the root and root canal anatomy of mandibular second molars with C-shaped root canal configurations in residents of the Hail district. The impact of gender and side on the frequency of root canal morphology was considered.

**Methods:**

The sample size for this study was 304 untreated mandibular second molars with completely developed roots on the right and left sides. Using CBCT on the teeth, the root form and canal morphology for each root are based on Vertucci's classification. The occurrence of canals in the shape of a C. The prevalence and resemblance of the left and right sides or men and females were investigated. The Chi-square test was performed to evaluate the findings.

**Results:**

Of the 304 mandibular second molars studied, 286 teeth had two roots (94.1%), whilst 13 (4.3%) were C-shaped root canal systems. 77 molars (25.3%) had two canal orifices, 219 (72.0%) had three canal orifices, and six (2.0%) and one (0.3%) had four and five root canal orifices, respectively. Type IV was the most common for mesial root, accounting for 57.7% of the sample (n = 176). For distal root, the most common occurrence was type I, which occurred 282 times (96.60%). The most prevalent root canal morphology was the presence of two canals in the mesial root and one canal in the distal root of teeth with two distinct roots (variant 3). (69.4%). The overall prevalence of C-shaped root canal systems is (4.3%) (n = 13).

**Conclusions:**

The patient's race is an undeniable factor that influences root canal anatomy. The root canal morphology of mandibular second molars revealed significant differences between Saudi subpopulations. The majority of mandibular second molars had two roots and three root canals. When treating these molars, the presence of a C-shaped root canal system must be taken into account.

## Background

A thorough cleaning, shaping, and three-dimensional obturation of the whole root canal system are critical to successful root canal therapy. To achieve these goals, a thorough understanding of root and canal anatomy is required [1]. Accurate knowledge of root and canal morphology is crucial for efficacious endodontic therapy [2]. A lack of such knowledge increases the possibility of missed root canals and procedural errors, which can lead to treatment failure [3]. Unfortunately, the architecture of the root canal system is highly complicated and variable [4], increasing the burden on dental practitioners to be conversant with such variances.

Numerous reports among various populations have analyzed mandibular molars and found a variety of structural differences and irregularities in their roots and canal systems [5–8]. Several studies [5–8] found that the root and canal morphology of mandibular second molars revealed a variety of complicated anatomical characteristics, which can complicate the phases of endodontic therapy [4]. One of the anatomical variants of mandibular second molars is the number of roots; while two roots are the most common. Single root, on the other hand, was found in 22–25% of Asian populations [6, 9] and 9–14% of Caucasians [10]. A third root located distolingually (radix entomolaris) or mesiobuccally (radix paramolaris) has been observed in 1.2% of Thais [6] 3.5% of Brazilians [11] and 3.45% of Turkish [12]. According to Mashyakhy et al. [13], most first and second mandibular molars have two roots and three canals, with the presence of a third root not uncommon. The canal configuration varies by population and is influenced by race, genetics, and ethnicity. Aldosimani et al. [14] found that the confluent type was the most common Mid Mesial Canal (MMC) configuration, followed by the fin-type, with no independent type found. The presence of MMC was unaffected by the patient's side, gender, or age.

Even with its frequency in a wide range of posterior teeth, including mandibular first premolars [15], maxillary first and second molars [16], the C-shaped canal morphology is most often seen in mandibular second molars. The major cause of C-shaped roots and canals is considered to be a failure of the epithelial root sheath to fuse on the root surface, either buccally or lingually [17]. The occurrence of a C-shaped canal was noticed to be 10% in African [5] and European populations [10], 6–44.5% in Eastern Asian populations [4, 6, 8, 9, 14, 18], 4.1% in a Turkish population [12], 10.6% and 9.1 in an Arab population [19, 20] and 3.5–10% in southern American populations [10, 11].

In general, the root canal morphology of mandibular second molars differs considerably amongst people and cultural groups all around the world. Some locations in Saudi Arabia investigated the root canal anatomy of mandibular second molars [13, 14, 19, 20]. However, there is no published study on the root and canal morphology of the mandibular second molars in Hail district. Therefore, the purpose of this study was to use CBCT to evaluate the root and root canal anatomy of mandibular second molars with C-shaped root canal configurations in Hail district residents. The impact of gender and side on the frequency of root canal morphology was considered.

## Method

The present study consisted of 304 CBCT images of mandibular permanent second molars that had been taken from patients who visited the Hail polyclinics dental Centre for diagnosis and preoperative assessment for nonsurgical and surgical endodontic treatment, dental implants, surgical extraction, and orthodontic treatment. This polyclinic is the largest in the Hail region. This center receives a large number of dental patients from all over the city and its surroundings. The Ethical Committee of the College of Dentistry at the University of Hail authorized this descriptive observational cross-sectional study (No: H-2021-025). Informed consent was waived by the ethics committee of the College of Dentistry, the University of Hail due to the retrospective nature of the study.

High-quality CBCT images were obtained between May 2018 and November 2020. Nonprobability purposive sampling was used in this study. A database including 3000 CBCT scans was examined. The CBCT images met the following inclusion criteria: clear CBCT scans of mandibular second molars having fully grown roots in people aged 18 to 65. Images of teeth restored with metallic restorations or with full coverage, or that caused artifacts in the scans or teeth treated endodontically or post-coronally were eliminated. Teeth associated with periapical disorders or that having resorption of root or calcification, as well as low-quality CBCT images, were also excluded. After analyzing 3000 images for inclusion/exclusion criteria, the final sample size for this study was 304 CBCT scans.

The scan was performed by using the Carestream CS 8100 3D (Carestream Dent LLC, Atlanta, USA) following the manufacturer’s recommended protocol. This machine's specifications were as follows: 60–90 kV, 2–15 mA, and 140 kHz, a CMOS sensor with Dental Volumetric Reconstruction (DVR), the scan time of 3–15 s, fields of view (FOV) of 4 × 4, 5 × 5, 8 × 5, and 8 × 8 cm, and voxel size of 75 m minimum. The images were analyzed using the CS 3D Imaging Software (Carestream Dent LLC, Atlanta, USA).

All examiners (M.I.A, S.M.A, A. S.A., and A.A.M) were calibrated prior to evaluation based on the criteria and variations specified prior to the experimental reading. The reliability of the interexaminer and intraexaminer was evaluated. The observers evaluated the images twice with a 2-week interval between assessments. The endodontists then simultaneously evaluated the CBCT image. The Kappa value for the intra-observer agreement was 0.90 for both observers and 0.86 for the inter-observer agreement. The examination of all mandibular second molars was performed under sagittal, axial, and coronal planes and the thickness slice was 0.3 mm. The contrast and brightness of the images' could be modified using software to guarantee excellent visibility.

The morphological characteristics recorded in the present study were as follows: the number of roots, their form, the number of canal offices, the type of root canals inside each root according to Vertucci's [1] classification, and major differences in root and canal anatomy according to Zhang et al. [9]. The occurrence of extra canals was also determined. According to Fan et al., C-shaped canals were also investigated. In addition, the curvature was determined according to the Schneider method [21]. The influence of gender and tooth position on the morphology were also recorded.

The Statistical Package for the Social Sciences, version 16.0, was used for statistical analyses (SPSS Inc., Chicago, IL, USA). The chi-square test was used to examine the relationship between root and canal morphology and the patient's gender and position sides. The significance level was set at *p *< 0.05 with a confidence interval (95%).

## Results

The number of roots on the right and left sides according to gender and tooth location were illustrated in Table 1. Of the 304 mandibular second molars studied, 286 teeth had two roots (94.1%), whilst 13 (4.3%) were fused roots. Fused roots were found in females (n = 7, 2.3%) more than males (n = 6, 2.0%). There was no statistically significant difference between groups in terms of sides or gender (*p *> 0.05).

**Table 1:** Root count for gender and tooth location

Number of roots	Gender			Tooth position		
	Male	Female	Total	Left side	Right side	Total
Fused roots n (%)	6 (2.0)	7 (2.3)	13 (4.3)	4 (1.3)	9 (2.9)	13 (4.3)
Two roots n (%)	134 (44.1)	152 (50)	286 (94.1)	146 (48.0)	140 (46.1)	286 (94.1)
Three roots n (%)	4 (1.3)	1 (0.4)	5 (1.6)	2 (0.7)	3 (1.0)	5 (1.6)

Chi-square, Fisher’s Exact tests; *p* > 0.05

As indicated in Table 2, 77 molars (25.3%) had two canal orifices, 219 (72.0%) had three canal orifices, and six (2.0%) and one (0.3%) had four and five root canal orifices, respectively. There were no significant variations in the number of root canal orifices between males and females, or between the right and left sides (*p *> 0.05).

**Table 2:** The number of canal offices for each gender and tooth location

Number of canal orifice	Gender			Tooth position		
	Male	Female	Total	Left side	Right side	Total
One-orifice n (%)	0 (0.0)	1 (0.3)	1 (0.3)	0 (0.0)	1 (0.3)	1 (0.3)
Two-orifice n (%)	33 (10.9)	44 (14.4)	77 (25.3)	40 (13.3)	37 (12.2)	77 (25.3)
Three-orifice n (%)	106 (34.9)	113 (37.2)	219 (72)	109 (35.9)	110 (36.2)	219 (72)
Four-orifice n (%)	4 (1.3)	2 (0.7)	6 (2)	1 (0.3)	5 (1.6)	6 (2.0)
Five-orifice n (%)	1 (0.3)	0 (0.0)	1 (0.3)	1 (0.3)	0 (0.0)	1 (0.3)

Chi-square, Fisher’s Exact tests; *p* > 0.05

Table 3 summarizes the presence of curvature in mesial or distal roots in relation to gender. The mesial root had mostly severe curvature of 177 (58.0%), however, in the distal root, the straight were found in 104 (35.6%) of the studied sample. There was no statistically significant difference between groups in terms of gender (*p *> 0.05).

**Table 3:** Location of curvature in the study sample

Variable	Mesial root			Distal root		
	Straight	Moderate	Severe	Straight	Moderate	Severe
Male	27 (8.9)	40 (13.1)	80 (26.2)	59 (20.2)	60 (20.5)	28 (9.6)
Female	13 (4.3)	48 (15.7)	97 (31.8)	45 (15.4)	75 (25.7)	25 (8.6)
Total	40 (13.1)	88 (28.9)	177 (58.0)	104 (35.6)	135 (46.2)	53 (18.2)

Chi-square, Fisher’s Exact tests; *p* > 0.05

**Fig. 1:**
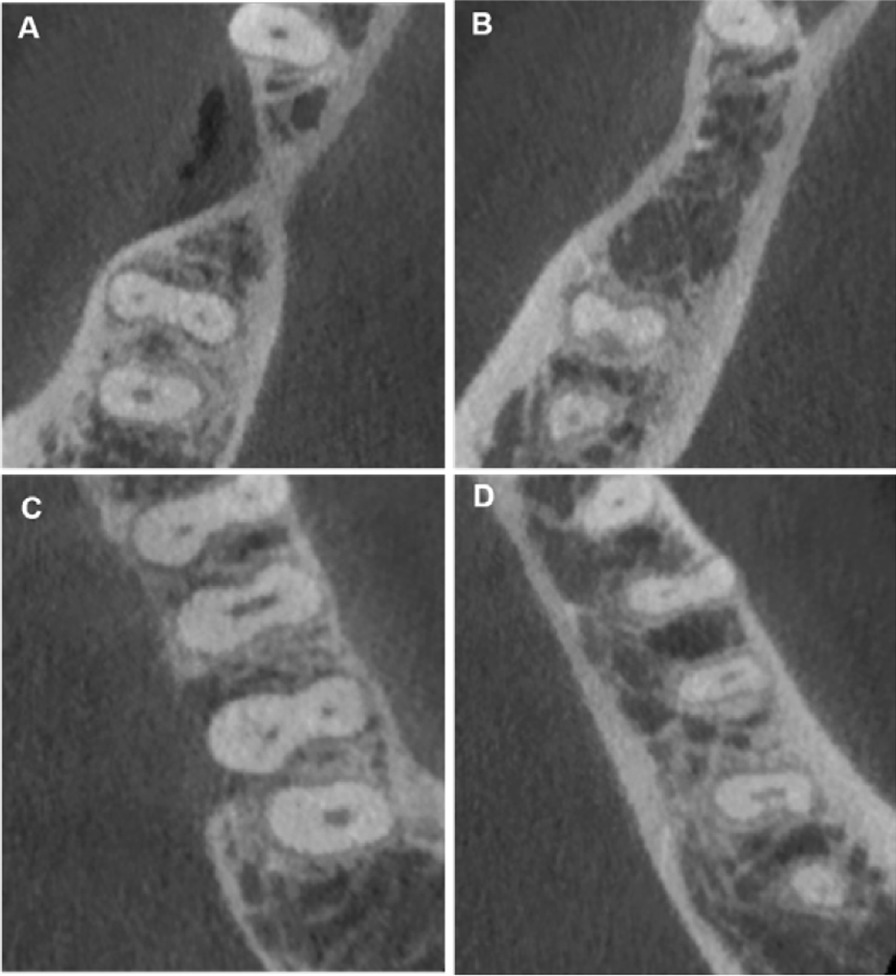
**A**, **B** Vertucci Type IV in mesial root and Type one in distal root. **C**, **D** Vertucci type II in mesial root

**Fig. 2:**
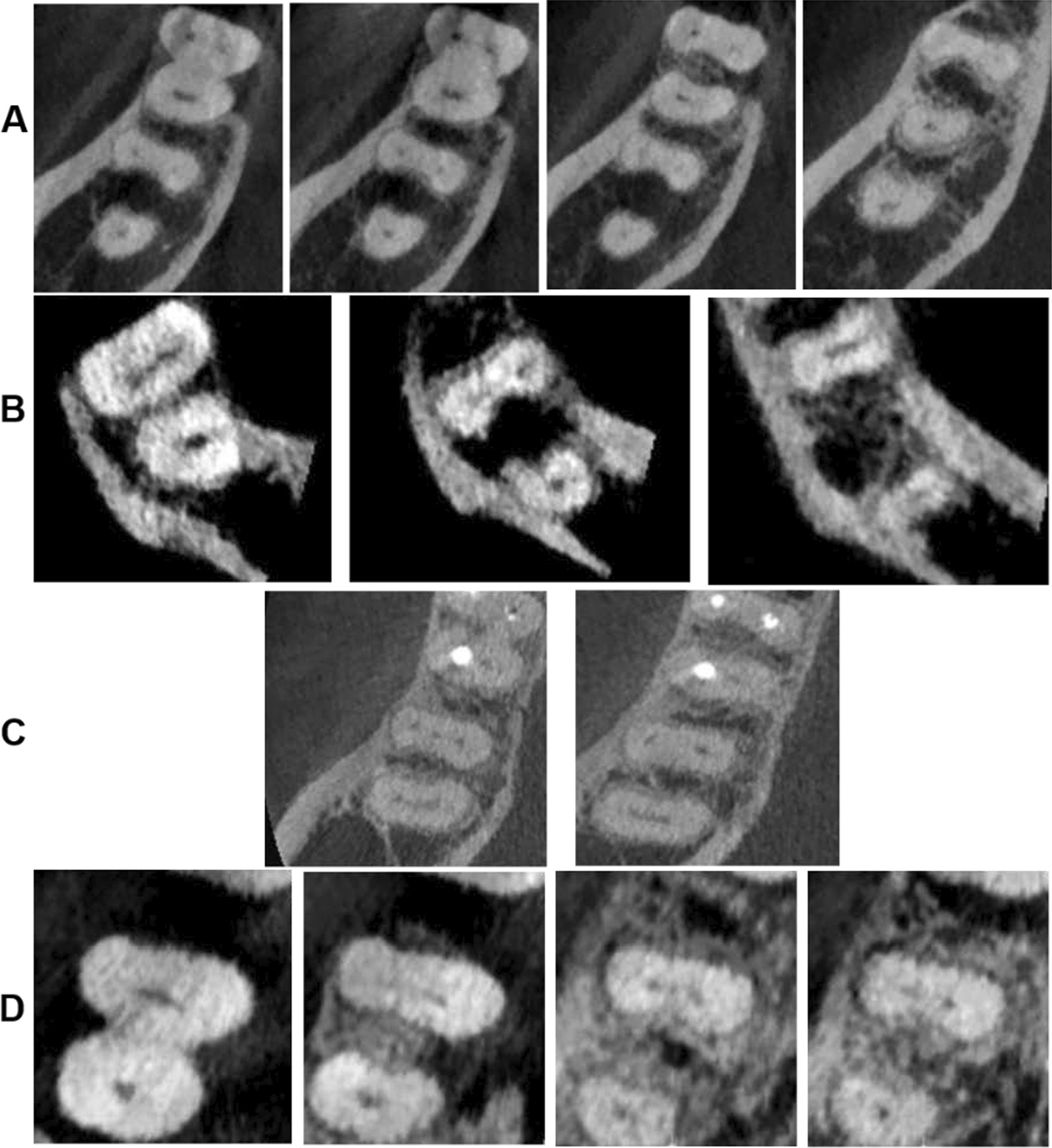
Some of the others variations. **A** 2-3-2-1 root canal configuration, **B** 3-2-1 canal configuration, **C** 3-2 configuration and **D** shows 1-2-3-2 canal configuration

This study found variations in root canal types according to Vertucci's classification (Fig. 1). Type IV was the most common for mesial root, accounting for 57.7% of the sample (n = 176), followed by type I (12.4%, n = 34) shown in Table 4. For distal root, the most common occurrence was type I, which occurred 282 times (96.60%), followed by Types IV (1.7%). The other variations that have not been recognized by Vertucci's classification were found in 0.7% of the specimens. Table 5 and Fig. 2 show the new variation that presents in the current study.

**Table 4:** Distribution of root canal types according to Vertucci’s classification

Type	1	2	3	4	5	Other variations
*Mesial root*						
Male	19 (6.2)	12 (3.9)	12 (3.9)	84 (27.5)	17 (5.6)	1 (0.3)
Female	19 (6.2)	17 (5.6)	14 (4.6)	92 (30.2)	14 (4.6)	3 (1.0)
Total	34 (12.4)	29 (9.5)	26 (8.5)	176 (57.7)	31 (10.2)	4 (1.4)
*Distal root*						
Male	143 (49.0)	1 (0.3)	0 (0.0)	2 (0.7)	0 (0.0)	1 (0.3)
Female	139 (47.6)	2 (0.7)	1 (0.3)	2 (0.7)	0 (0.0)	1 (0.3)
Total	282 (96.6)	3 (1.0)	1 (0.3)	5 (1.7)	0 (0.0)	2 (0.7)

Chi-square, Fisher’s Exact tests; *p* > 0.05

**Table 5:** New root canal types found the study sample

	1-2-3-2	3-2-1	3-2	2-3-2-1
Male	1 (0.3)	1 ( 0.3)	0 (0.0)	0 (0.0)
Female	0 (0.0)	1 (0.3)	1 (0.3)	2 (0.7)
Total	1 (0.3)	2 (0.7)	1 (0.3)	2 (0.7)

Variants 1, 3, 4, 5, 8, 9, 10, and 11 were reported in this investigation, according to Zhang et al. [9] as shown in Table 6. The most prevalent root canal morphology was the presence of two canals in the mesial root and one canal in the distal root of teeth with two distinct roots (variant 3). (69.4%). Variant 1 is presented in 22.4%, followed by variant 9 (3.0%), variant 4 (2.0%) and variant 10 (0.7), while either variants 8 or 11 were presented in 0.3% as shown in Table 6.

**Table 6:** Variants of root and canal system morphology according Zhang et al.

Variant	1	3	4	5	8	9	10
Male	29 (9.5)	100 (32.9)	4 (1.3)	5 (1.6)	0 (0.0)	4 (1.3)	1 (0.3)
Female	39 (12.8)	111 (36.5)	2 (0.7)	1 (0.3)	1 (0.3)	5 (1.6)	1 (0.3)
Total	68 (22.4)	211 (69.4)	6 (2.0)	6 (2.0)	1 (0.3)	9 (3)	2 (0.7)

Chi-square, Fisher’s Exact tests; *p *> 0.05

**Fig. 3:**
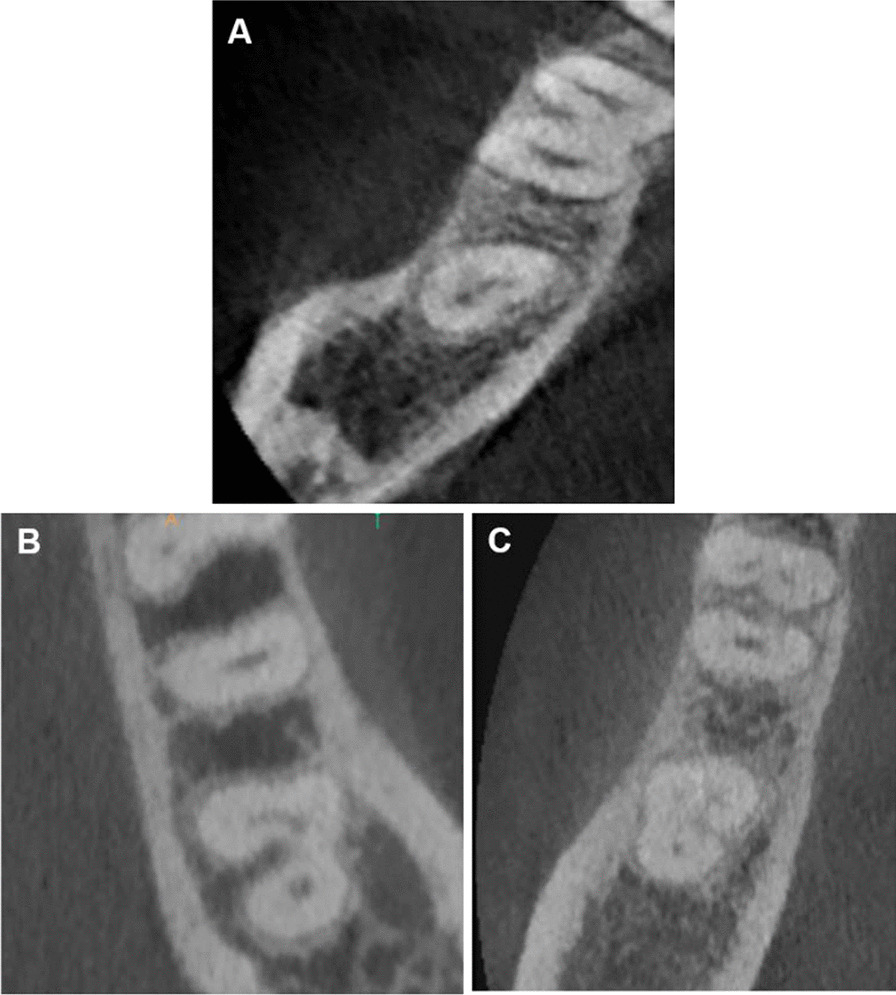
According to Fan classification of C-shaped root canals, **A** type C1, **B** C2 and **C** C3c

The overall prevalence of C-shaped canals is (4.3%) (n=13) as shown in Table 6. Either C2 or C3 represented the majority of canals (46.1%). The minority of canals (7.7%) demonstrated an uninterrupted C-shape (C1) as shown in Table 7 and Fig. 3.

**Table 7:** Prevalence of C-shaped canals according Fan classification

	C1	C2	C3
Male	0 (0.0)	4 (30.7)	3 (23.0)
Female	1 (7.7)	2 (15.4)	3 (23.0)
Total	1 (7.7)	6 (46.1)	6 (46.1)

Overall prevalence of c-shaped canals is (4.3%) (n = 13)

## Discussion

Numerous investigations and analyses of various teeth across different people have clearly proven anatomical differences in exterior and interior tooth morphologies related to ethnicity. Several studies throughout the world have identified possible variations in the exterior and interior anatomies of mandibular first molars based on race and geographical area, with varying percentages of each anatomical characteristic [22–27]. The current study is the first to investigate and characterize the root structure and root canal morphology of mandibular second molars in the Hail area of Saudi Arabia.

Many techniques have been used to determine the morphologic features of the root canal system. Despite the fact that the clearing approach has been utilized for a long time, it is a destructive and non-reproducible procedure that can only be employed in vitro and may generate artifacts when used to determine the root canal architecture [28]. The staining method might not adequately replicate the true root canal anatomy Attributable to the inability of dye to penetrate and stain the whole root canal system in cleaned teeth; particularly in Vertucci type I canal design [28]. Despite the fact that micro-CT has overcome these limitations by giving comprehensive qualitative and quantitative measurements of root canal morphology involving minor anatomical details such as accessory canals, foramina, apical delta, and isthmi, micro-CT is not widely available around the world, particularly in developing countries. Additionally, its greater cost, radiation dosage, and longer exposure time are all characteristics that limit its usage to in vitro only [28]. Because of the significant information gained from its coronal, sagittal, and axial plans, CBCT was chosen as the evaluation technique in this study for the assessment of root and root canal architecture of mandibular first molars. It offers doctors a viable device for noninvasive and three-dimensional reconstruction imaging in tooth morphological examination and other applications during endodontic treatment [29–31]. When compared to previous techniques, CBCT pictures facilitated the evaluation of molars without causing tooth loss. When compared to conventional procedures such as clearing and micro-CT techniques, CBCT has demonstrated its dependability and accuracy in showing the number and location of root canals. Furthermore, CBCT is a readily available and less costly technology that may be employed in vivo or ex vivo. Other benefits of CBCT include reduced radiation dosage, no superimposition of anatomical features, and less picture distortion [28].

In the current study, Vertucci's classification [32] was chosen as a reference for canal types since it was the first system to detect more complicated canal system configurations than prior classifications. Despite the fact that it has been a fundamental categorization for a long time, it is still frequently employed in new research in the literature by most authors [28, 33], and it was utilized in the current study for easy comparison with the results of other investigations. Furthermore, the Zhang et al. [9] classification was chosen in this study because it is a complete classification that relates the number of roots to the number of root canals in each tooth and provides a straightforward description of tooth internal and exterior anatomy.

The majority of mandibular second molar teeth (94.1%) had two roots, which was consistent with earlier research from diverse groups [13, 34, 35]. This is with the same line to the findings found in Turkish (85.4%) [12], Indians (88.8%) [36], Belgians (83.93%), and Chileans (86.61%) [10]. Furthermore, 3-rooted molars were found in (1.6%), which was consistent with a previous study on the Saudi population (1.48% and 1.7%) [13, 19, 24], but was greater than that found in Belgians (0.89%) [10] and Koreans (0.3%) [37]. However, larger numbers were found in Turkish (3.45%) [12], Brazilians (3.5%) [11], and Chileans (3.57%) [10]. Despite the modest proportion of additional roots identified in this report, it should be predictable and searched for as a type of unique root disparity in these molars [38]. Applying periapical radiographs with different angles or CBCT aids in the evaluation of tooth architecture, preventing missing canals and eventual treatment failure.

Because there is some link between root growth and the X chromosome, dental anatomy studies generally include a gender characterization. This investigation showed no statistically significant gender differences in the prevalence of the number of molar roots, which was consistent with the findings of the Turkish study by Demirbuga et al. [12]. In this study, 4.3% of mandibular second molars had fused roots, which is close to the 8.97% described in Turkish [12]. However, larger numbers (24 and 39%) were reported in Chinese [9, 18].

In this study, mandibular second molars with three orifices were the most prevalent (72%) followed by two orifices (25.3%). This is consistent with findings in Turkish people (72.8% with three orifices and 22.8% with two) [12] and Chinese populations (46% with three orifices and 38% with two) [9]. In the same line, mandibular second molars with three canal orifices were the most common (77%, n = 385), followed by two canal orifices (21%, n = 105) were reported in Yemeni populations [35]. There were no significant variations in the number of root canal orifices between males and females, or between the right and left sides (*p *> 0.05). These results are in agreement with findings in Yemeni populations [35].

To avoid unforeseen occurrences during root canal therapy, the doctor should thoroughly examine all root canal curvatures. The mesial root showed greater curvature in the current study, which agrees with Peters [39], who reported that the apical anatomy of mesial roots displayed morphological complexity. They also said that the curvatures caused asymmetric dentin loss after cleaning, resulting in apical transport.

The mesial root of mandibular second molars revealed more type IV. This corresponds with findings in Sudanese [5], Chinese [9], and Turkish [12], where mesial roots mostly possessed type IV canals but differ from findings in Yemen [35]. Our findings similarly contradict those in Belgians and Chileans [10], where type III canals were the most prevalent, followed by type V. We found that the distal root had predominantly type I canals, which was similar to Thai [6], Sudanese [5], Chinese [9], Turkish [12], Belgians, and Chileans [10]. In 1.4% of the mesial roots and 0.7% of the distal roots, root canal types 1-2-3-2, 3-2-1, 2-3-2-1, and 3-2 were found. It is classified as a non-classifiable Vertucci type. The categorization formula proposed by Ahmed et al. [40] may be used to characterize this arrangement, which describes the tooth number, the number of roots, and the canal type in each root.

Of the 304 mandibular second molars studied, 211 (69.4%) had two distinct roots with three canals (variant 3). The present finding was consistent with earlier reports [41, 42]. Moreover, Zhang et al. [9] reported that the mandibular second molars had the majority of two roots and three canals [9].

Martins et al. [43] reported that gender and geographic region might be confounding factors for the prevalence of C-shaped anatomy in mandibular second molars, whereas age had no effect on the prevalence of C-shaped anatomy in this tooth group. Knowledge of these preoperative factors, combined with the use of an appropriate diagnostic tool, would assist clinicians in anticipating and treating this complex morphological variation of root canals in practice. Using CBCT technology, von Zuben et al. [44] compared the prevalence of C-shaped mandibular second molars in different parts of the world. They reported that the prevalence of C-shaped anatomy varied by region. They found that China had the highest prevalence (44.0%), while Brazil had the lowest (6.8%). The findings from China were considerably higher than in any other region. C-shaped canals were found in 4.3% of mandibular first molars in this research, which is comparable to Brazilians (3.5%) [11] and Turkish (4.1%) [12]. The current investigation found a lower prevalence than a previous study in Saudi Arabians (9.1%) [13, 24]. However, it was much lower than the findings obtained by Lebanese (19.1%) [45], Chinese (29%, 38.6%) [9, 18], Koreans (39.8%) [8], and Malaysians (48.7%) [46]. In 4.4% of cases, the canal structure remains constant from orifice to apical level. Similar observations were found in Iranians [7] and Chinese [18], where 4.9% and 5.9% of C-shaped canals, respectively, remained constant along the root length. This is consistent with the findings of Fan et al. [47], who reported that the form and quantity of C-shaped canals change throughout the root length. As a result, the form of the canal orifice cannot be used to predict the C-shaped canal architecture along the tooth root to the apex.

C-shaped canals are complicated and uneven areas with potentially diseased soft-tissue remnants or debris that may evade conventional cleaning and filling operations [48]. As a result, when C-shaped canals are found, they may be carefully debrided and obturated to enable effective root canal therapy. The access cavity and obturation for teeth with a C-shaped root canal system vary greatly and are determined by the pulp architecture of the individual tooth. Alternative canal cleaning procedures, such as those utilizing ultrasonics, would be more successful in general; an increased volume of irrigant and deeper penetration with tiny instruments utilizing sonics or ultrasonics allows for higher cleanability in fan-shaped regions of the C-shaped canal. The mesial and distal canal spaces can be prepared and obturated as normal canals; however, sealing the buccal/lingual isthmus is problematic if lateral condensation is the sole approach utilized; hence, application of thermoplasticized gutta-percha is more suited [48].

It is critical to consider and identify this difference in order to ensure successful endodontic therapy. Before beginning endodontic treatment, a careful examination of radiographs taken from various angles is required to increase the likelihood of detecting such anatomical variations and reducing the risk of missing a canal; additionally, CBCT is recommended in patients suspected of having an additional root. To avoid problems, if an additional root is found, the patient should be referred to an endodontist.

The current study represented the internal root anatomy of mandibular second molars in Saudi residents and provided a theoretical foundation for clinical care to some extent. The results of anatomical forms of root canals were heavily influenced by sample size and experimental approach. However, there are a few drawbacks that must be addressed. Because this was a single-center study, the sample size should have been larger. Moreover, this study is retrospective inconsistent scans voxel and field size, since may affect the readings. Multicenter studies with a larger sample size may provide a more accurate estimate of the prevalence of this anomaly in the Saudi population. Furthermore, the spatial resolution of the CBCT used in this study was lower than that of micro-and nano-CT, which could have influenced the results.

## Conclusions

Under the limitations of the current study, it can be concluded that the patient's race is an undeniable factor that influences root canal anatomy. The root canal morphology of mandibular second molars revealed significant differences between Saudi subpopulations. The majority of mandibular second molars had two roots and three root canals. The presence of C-shaped root canals must be considered when treating these molars. This anatomical difference must be found in order for root canal therapy to be successful.

## Data Availability

The datasets generated and/or analyzed during the present study are not publicly available as ethics approval was granted on the basis that only the researchers involved in the study could access the identified data but are available and accessible from the corresponding author on reasonable request.

## Abbreviations

3D: Three dimension
MMC: Mid Mesial Canal
FOV: Fields of view
CBCT: Cone beam computed tomography
DVR: Dental volumetric reconstruction
